# Anti-Viral Therapy and Decreased Sexual Desire in Patients with Chronic Hepatitis C

**DOI:** 10.1371/journal.pone.0160450

**Published:** 2016-08-09

**Authors:** Po-Jen Hsiao, Po-Fan Hsieh, Eric Chieh-Lung Chou, Hsueh–Chou Lai, Cheng-Yuan Peng, Kuan-Pin Su

**Affiliations:** 1 Department of Urology, China Medical University Hospital, Taichung, Taiwan; 2 School of Chinese Medicine, China Medical University, Taichung, Taiwan; 3 Graduate Institute of Clinical Medical Science, Taichung, Taiwan; 4 Division of Hepato-gastroenterology, department of internal medicine, China Medical University Hospital, Taichung, Taiwan; 5 School of Medicine, China Medical University, Taichung, Taiwan; 6 Department of Psychiatry, & Mind-Body Interface Laboratory (MBI-Lab), China Medical University Hospital, Taichung, Taiwan; National Taiwan University Hospital, TAIWAN

## Abstract

**Purpose:**

Peg-interferon (PegIFN)α2a or PegIFNα2b plus ribavirin (RBV) is the standard therapy for chronic hepatitis C virus (HCV) infection in Taiwan and Asia. It is commonly associated with adverse effects, but the issue of sexual and mental health is not well reported. This study aimed to evaluate the impact of anti-viral therapy with PegIFNα plus RBV on sexual desire and depression.

**Methods:**

This prospective cohort study from 2009 to 2014 enrolled 181 patients with HCV who received PegIFNα2a (180 mcg/week) or PegIFNα2b (1.5 mcg/Kg/week) plus RBV (800–1200 mg/day) according to response-guide therapy for 24 to 48 weeks in a tertiary medical center. Patients with decreased sexual desire (DSD) before PegIFNα plus RBV were excluded. Patients were evaluated at baseline (week 0) and after 2, 4, 8, 12, 16, 20, and 24 weeks of PegIFNα plus RBV treatment using the structured Mini-International Neuropsychiatric Interview, for the diagnosis of a major depressive episode, and the 21-item Beck Depression Inventory (BDI), for monitoring depressive symptoms. The 21st item of the BDI was used to evaluate DSD.

**Results:**

During therapy, 124 (68.5%) patients had DSD. The BDI score peaked at 14.8 weeks. The severity of DSD was greatest at 16 weeks of treatment. The average score of the 21st item of the BDI correlated with DSD. Depression history and the prevalence of subsequent major depressive disorder after anti-viral therapy was correlated to DSD (p = 0.05 and 0.001). Male patients complained of DSD more significantly than females (p = 0.031).

**Conclusions:**

Decreased sexual desire is common but is usually neglected in patients with chronic hepatitis C undergoing anti-viral therapy, especially among male patients. Physicians must be monitoring the side effects of sexual health and depression.

## Introduction

The prevalence of chronic hepatitis C virus (HCV) infection is approximately 3% worldwide and it is more common in Asia than in America or Europe [1]. In Taiwan and most areas of Asia, Peg-interferon plus ribavirin (PegINF+RBV) is currently the standard therapy [2]. It is associated with some adverse effects, including fatigue, muscle aches, headaches, gastrointestinal symptoms, bone marrow effects, and psychological difficulties like anxiety, depression, and difficulty sleeping [3]. However, the issue of sexual health impairment is not well reported in anti-viral treatment. The aim of this study was to evaluate the impact of anti-viral therapy on sexual desire and depression.

## Materials and Methods

The study protocol conforms to the ethical guidelines of the 1975 Declaration of Helsinki. The protocol was approved by the Research Ethics Committee of China Medical University Hospital (IRB Number:CMUH102-REC2-038). Written informed consent was obtained from patients for both the provided treatment as well as for the use of their data in this specific study.

### Patients

**Fig 1:**
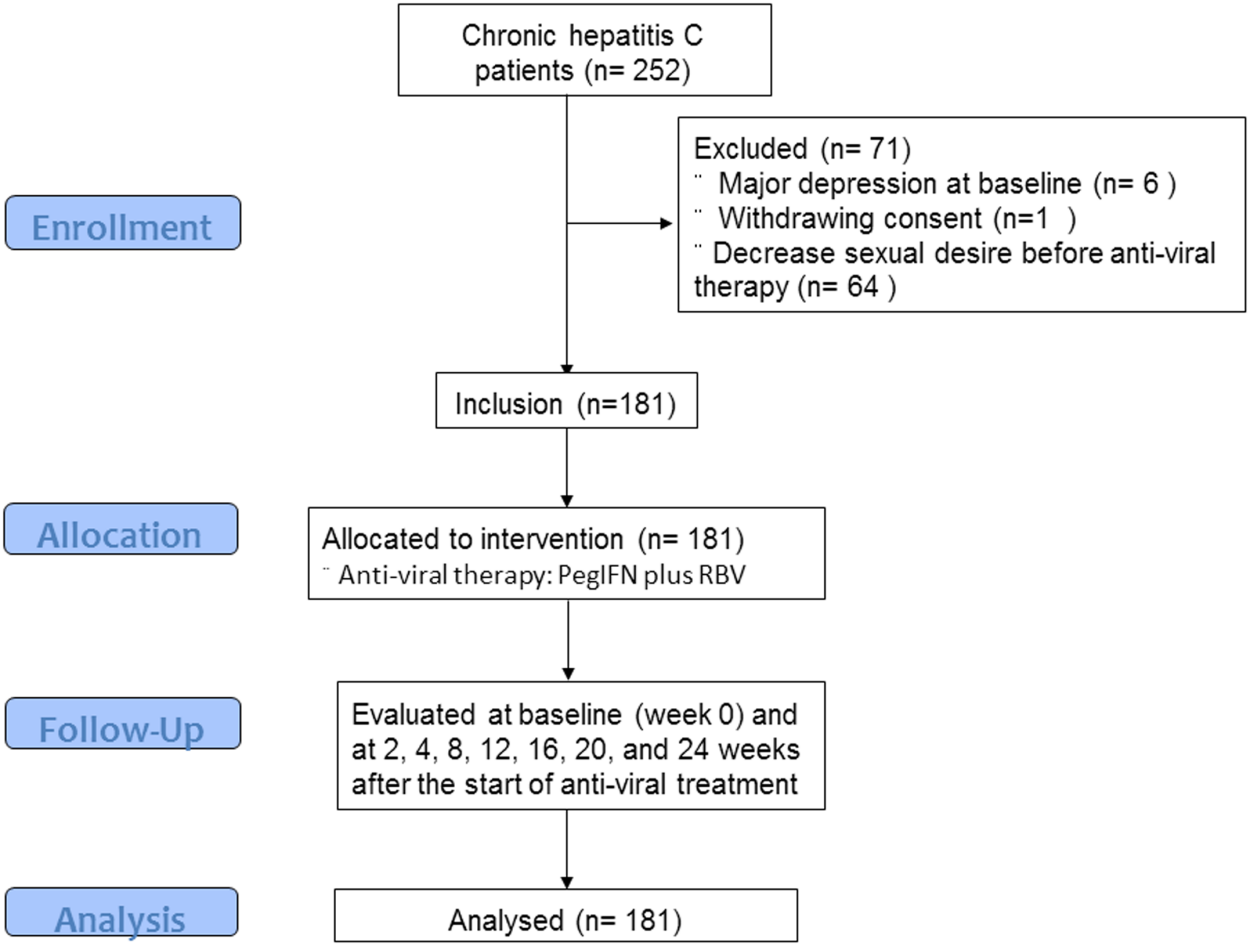
Flow chart of included patients in the study.

This single institutional prospective study enrolled patients diagnosed with chronic hepatitis C at a tertiary referral center and who were undergoing the combination therapy with PegIFNα2a (180 mcg/week) plus RBV (800-1200mg/day) or PegIFNα2b (1.5 mcg/Kg/week) plus RBV. We treated patients with chronic hepatitis C based on The Taiwan National Health Insurance drug reimbursements in 2009. According to Taiwan National Health Insurance payment for chronic hepatitis C, antiviral therapy was in accordance with response-guide therapy regardless of genotype. The treatment was 24 weeks for chronic hepatitis C patients who received peg-interferon plus ribavirin therapy with rapid virologic response (RVR), 48weeks for those with an early virological response (EVR) and early terminated in those without an EVR. During treatment, they were evaluated at baseline (week 0) and at 2, 4, 8, 12, 16, 20, 24 weeks, and on-demand after the start of anti-viral treatment. On each visit, the patients were assessed by a psychiatrist (K.P. Su) using the structured Mini-International Neuropsychiatric Interview (MINI) to ascertain the diagnosis of a major depressive disorder episode, and by the Beck Depression Inventory (BDI) to monitor depressive symptoms [4]. The 21st item of the BDI was used for evaluating decreased sexual desire (DSD). Patients were excluded if they had major depression or DSD at baseline, withdrew from the study, or were lost to follow-up. Those taking anti-depressants, mood stabilizers, anti-psychotics, or substance abuse were also excluded (Fig 1). All of the patients had written informed consent before treatment.

### Statistical Analysis

The independent t test was used to compare means of continuous variables between case and control subjects. Changes in sexual desire levels as a function of time were assessed using repeated-measures analysis of variance (ANOVA) with mixed linear modeling. All comparisons of sex, marriage status, and depression history were conducted using the chi-square test, with Yates’ correction when appropriate. All probabilities were two-tailed. Statistical significance was set at p<0.05. All analyses used the SPSS statistical software version 17 (SPSS Inc., Chicago, Illinois, USA).

## Results

A total of 252 patients were enrolled. After excluding six for major depression at baseline, one for withdrawing consent and 64 for DSD before anti-viral therapy, 181 patients were included (Table 1). Their mean age was 48.9 years. There are 110 males and 71 females, and most (83%) were married.

**Table 1:** The sociodemographic and clinical data of the study patients.

Characteristics	n = 181
Age, years (mean ±SD)	48.91±11.19
Sex (Male/Female, n)	110/71
Education (years)	9.85±4.12
Marriage, n	
*Married*	150
*Unmarried (Single/divorced/widow or widower)*	31 (15/11/5)
DSD	124 (68.5%)
Non DSD	57 (31.5%)
Depression history	6 (3.3%)
Major depression episode	52 (28.7%)
PegINF2a	110(61%)
PegINF2b	71(39%)

Abbreviation: DSD, Decreased sexual desire (21-item BDI >0); BDI, Beck Depression Inventory

After receiving PegIFN plus RBV (PegIFN+RBV) treatment, 124 (68.5%) patients had DSD. Fifty-two (28.7%) developed major depression during treatment. 110 patients treated with PegIFNα2a plus ribavirin and 71 patients received the PegIFNα2b plus ribavirin therapy, respectively.

**Fig 2:**
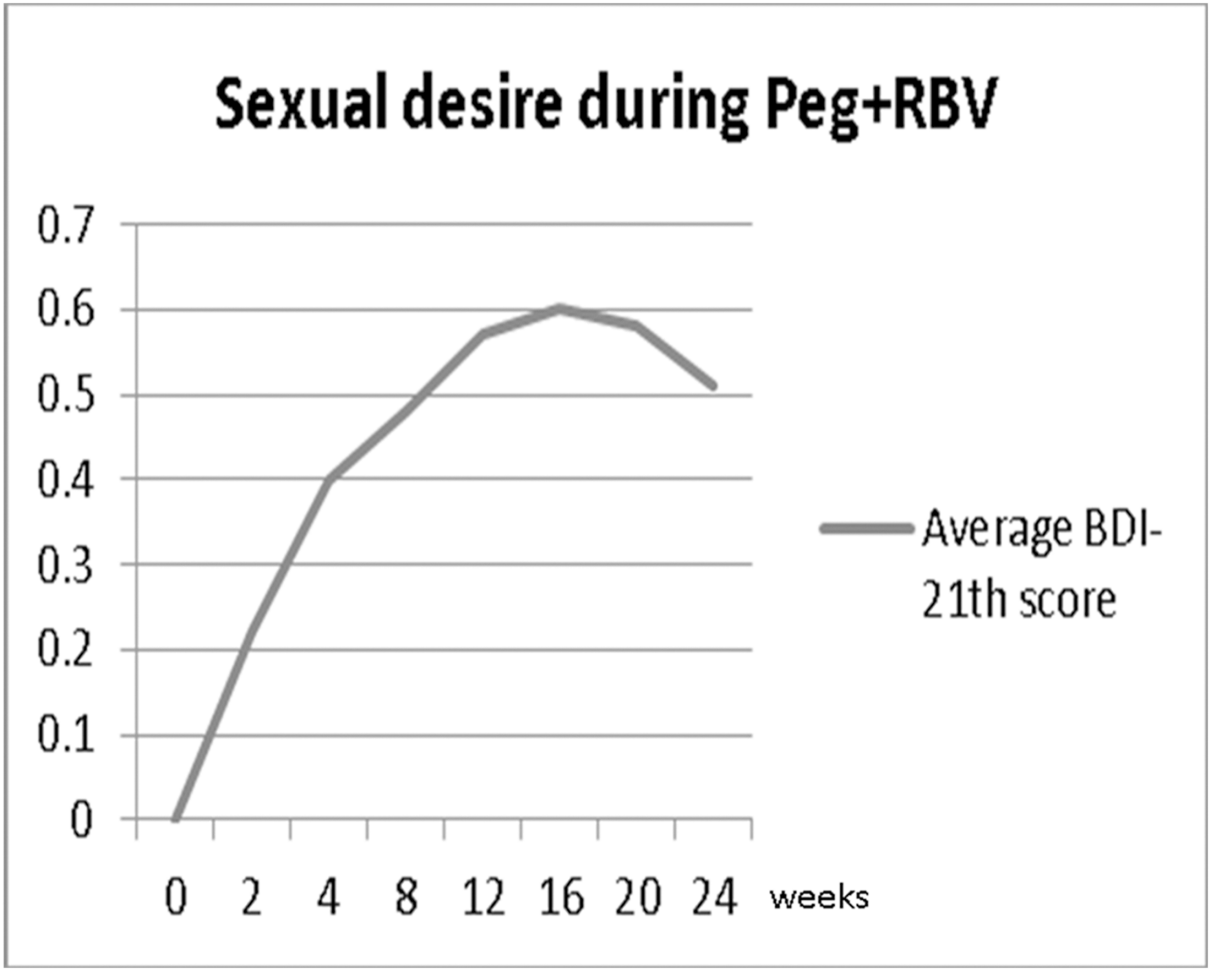
Time course of the 21-item Beck Depression Inventory (BDI) scores and BDI scores of decreased sexual desire in hepatitis C patients before (week 0) and during anti-viral therapy. The higher values represented greater deterioration.

The severity of DSD was greatest after 16 weeks of treatment (Fig 2). Major depressive disorder, as assessed by the average score of BDI, peaked at 14.8 weeks. There were no statistical differences in age, education level, and marital status between patients with and those without DSD during treatment (Table 2). Six patients had depression history and all of them suffered DSD after anti-viral therapy (p = 0.05). The prevalence of subsequent major depressive disorder after anti-viral therapy was higher in patients with DSD (37.1% vs. 10.5%, p = 0.001). The frequency of depression during treatment in patients treated with PegIFNα2b plus ribavirin (41%) was significantly higher than those treated with PegIFNα2a plus ribavirin (21%, p = 0.004), however, there was no statistical significant difference in decreased sexual desire (DSD) between patients treated with PegIFNα2b and PegIFNα2a plus ribavirin (p = 0.271).

**Table 2:** Comparison of patients with or without DSD during PegIFN+RBV treatment.

	With DSD(n = 124)	Without DSD (n = 57)	*p* value
Age, years (mean±SD)	49.38±10.13	47.89±13.23	0.407
Sex			0.031
*Male*	82	28	
*Female*	42	29	
Education (years)	9.60±3.71	10.39±4.88	0.232
Marriage			0.587
*Married*	103	47	
*Unmarried*	21	10	
Depression history	9	0	0.050
Depression during antiviral therapy	46 (37.1%)	6 (10.5%)	0.001
PegIFN type			0.271
*PegIFNα2a*	72(58%)	38(67%)	
*PegIFNα2b*	52(42%)	19(33%)	

Abbreviations: DSD, Decreased sexual desire (21-item BDI >0); Without DSD, no decreased sexual desire (21-item BDI of 0); BDI, Beck Depression Inventory; PegIFN, Peginterferon; SD, standard deviation. The *p* values were from results of independent-samples t test and x^2^ test

The baseline BDI score was higher in females (3.00±3.90 vs. 4.59±5.94, p = 0.035). Male patients developed DSD more often than females (74.5% vs. 59.2%, p = 0.03) (Table 3). On the other hand, female patients suffered from more major depressive episodes than males (38% vs. 22.7%, p = 0.03).

**Table 3:** Characteristics of the study patients, by sex.

	Male (n = 110)	Female (n = 71)	p value
Age, years (mean ± SD)	48.66±10.51	49.30±12.22	0.710
Education (years)	10.27±3.50	9.18±4.89	0.084
Marriage			
*Married*	97	53	0.118
*Unmarried*	13	18	0.118
Depression history	3 (2.7%)	3 (4.2%)	0.585
Depression during antiviral therapy	25 (22.7%)	27 (38.0%)	0.028
DSD	82 (74.55%)	42 (59.15%)	0.031
PegIFN type			0.196
*PegIFNα2a*	71(65%)	39(55%)	
*PegIFNα2b*	39(35%)	32(45%)	

Abbreviations: DSD, Decreased sexual desire (21-item BDI >0); Without DSD, no decreased sexual desire (21-item BDI of 0); BDI, Beck Depression Inventory; PegIFN, Peginterferon; SD, standard deviation. The *p* values were from results of independent-samples t test and x^2^ test

In our study, 143 patients had record the data of treatment of response. 106 patients (74.1%) achieved a sustained virologic response (SVR) with negative HCV PCR results 24 weeks after completion of antiviral therapy and 37 patients (25.9%) did not achieved SVR. There was no statistical significance between SVR and decrease sexual desire (P = 0.948).

## Discussion

The prevalence of HCV infection is higher in countries in the east than those in the west [5]. Among those with chronic HCV infection, 30% will advance to liver cirrhosis and liver cancer, which is the leading cause of death for patients with hepatitis C in Asia. PegIFN plus RBV therapy according to response-guide therapy for 24 to 48 weeks is the standard anti-viral therapy in Asia [6]. However, the sequelae of PegIFN plus RBV therapy is an another healthcare problem.

Many side effects of PegIFN plus RBV therapy, such as nausea, recurrent vomiting, fatigue, and mood changes including depression, are well-reported [7,8]. Previous studies show that the incidence of major depression during PegIFN plus RBV therapy is 26.5% [9]. However, there has been no well-designed study on DSD and the correlation between depression and DSD. In the present study, 28.7% patients developed major depression disorder during the anti-viral treatment, while 68.5% patients suffered from DSD.

**Fig 3:**
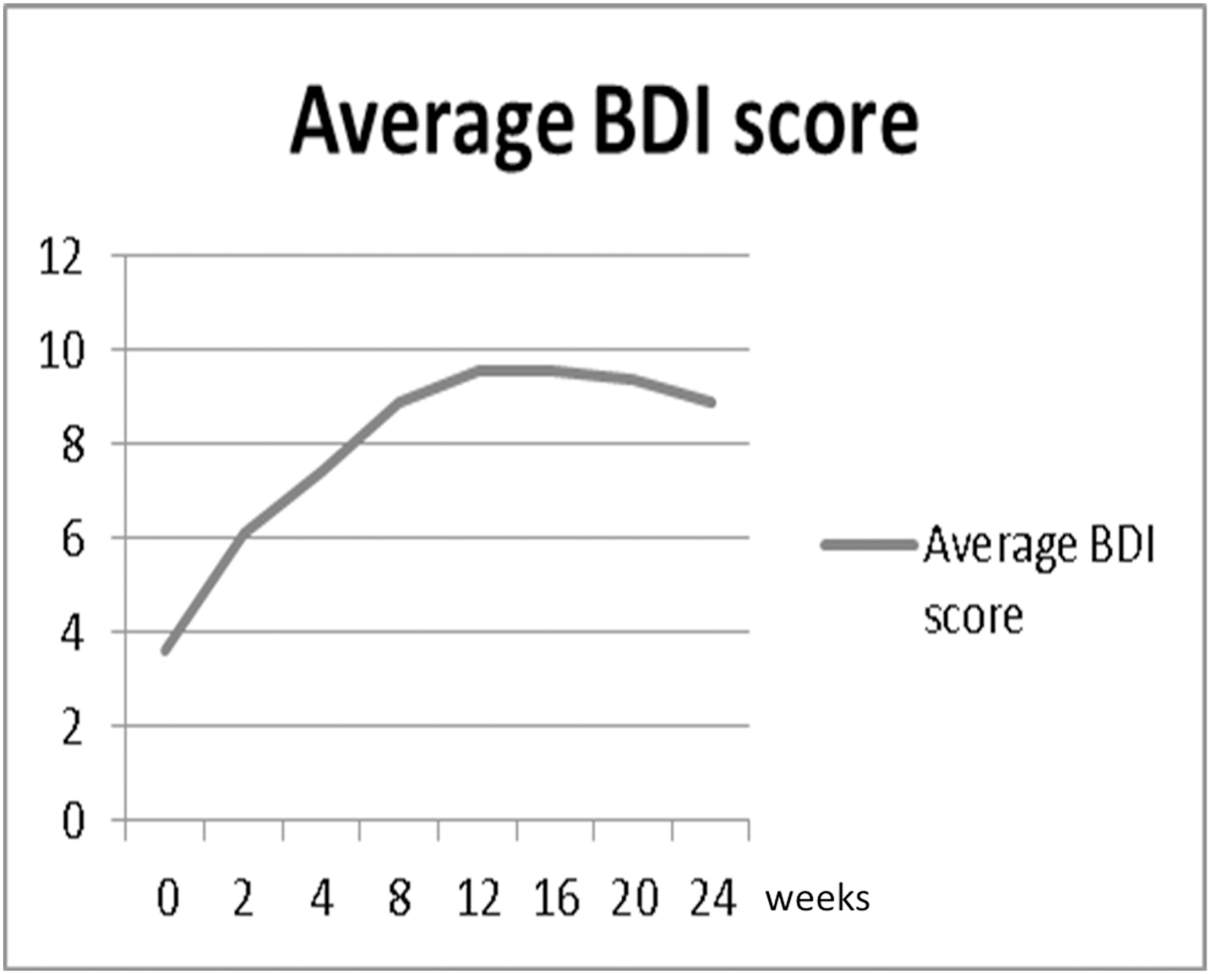
Time course of the total Beck Depression Inventory (BDI) scores in hepatitis C patients before (week 0) and during anti-viral therapy. The higher values represented greater deterioration.

Many studies demonstrate that PegIFN plus RBV may cause depression and mood disorders. Guy Bodenmann et al. reported an association between depressed mood and sexuality [10]. Another case control study also found a relationship between depression and DSD [11]. In the present study, the peak of BDI score (14.8 weeks) was very close to that of DSD (Fig 3). Some studies reported that depression symptoms remained through the 14th and 16th weeks of treatment [12].

The prevalence of subsequent major depressive disorder during PegIFN plus RBV therapy is higher in patients with DSD than in those without DSD (37.1% vs. 10.5%, p = 0.001). The correlation between DSD and depression may be explained by the assumption that if patients have depressive mood, they may easily lose their sexual desire. Moreover, all patients with depression history suffered from DSD after PegIFN plus RBV (p<0.001). It is possible that patients with depression may have unstable psychological status and tend to develop DSD during PegIFN plus RBV.

In this study, the prevalence of DSD is higher than that of depression during PegIFN plus RBV. Nonetheless, DSD is not only induced by depression and other factors may also play a role. PegIFN plus RBV therapy can cause not only physiologic but also psychological effects. Literature indicates that PegIFN plus RBV can change serum levels of sex hormone such as testosterone, gonadotropins (LH, FSH), prolactin, DHEAS, and estrogen which were related to sexual desire [13–16].

There is a discrepancy that patients with decreased sexual desire were male predominant and had more depression during therapy (Table 2), but less depression during therapy were found in males than in females (Table 3). First, sex hormones influence males more than females due to effects on the male testes, resulting in decreased male libido [17,18]. Some studies assume that estrogen has minimal effect on libido and point out that estrogen level is not significantly changed in PegIFN plus RBV therapy [19]. In this study, PegIFN plus RBV has less effect on female’s libido than on men’s. In other words, males more frequently develop DSD than females (p = 0.03). Second, some scholars agree with the theory of increasing indoleamine (2,3) -dioxygenase activity which will cause the degradation of tryptophan (the precursor of serotonin (5-HT)) in brain [20]. When patients had insufficient serotonin, they will tend to depression. Men have higher serotonin level in their brain. Therefore, the impact of the drug related serotonin insufficiency may be lesser than women. Third, the proportion of depression in men is fewer than women in the general population. Because men are less likely affected by environmental factors or emotion, women pay more attention to the environment and other people's perception. In addition, there are some studies suggest that depressive gene is dominant and located on x chromosome which is double set in women and single in men [21]. Therefore, women are likely to exhibit depression.

Dove et al. report that male American patients have impaired sexual desire and satisfaction during anti-viral therapy [22]. Overall, 39% of participants described impaired sexual desire at baseline, but this percentage increases to 54–56% during the remainder of treatment [19]. In this study, 26% of patients complained of DSD before treatment. The psychology and sexual character of Americans are quite different from those of the Chinese. The Chinese may feel shy while talking about sexual activity so the prevalence of DSD may be underestimated.

During treatment, male Chinese patients have a higher proportion (74.55%) of DSD than American patients. One possible reason is those Dove’s study did not exclude anti-depressant users [22]. Another reason is that the divorce rate is higher in America and widows and widowers may neglect sexual desire because of the lack of a partner. Lastly, the genotypes of drug reaction are different between Americans and Asians [23].

Patients who achieved SVR have significantly fewer complications related by liver, less hepatocellular cancer, and fewer liver-related deaths [24]. But there is still lack of studies in the association of on-treatment response with decreased sexual desire.

The type of PegIFN may cause mood problems. The frequency of depression during treatment in patients treated with PegIFNα2b plus ribavirin was significantly higher than those treated with PegIFNα2a plus ribavirin. Fried MW et al. also showed patients treated with PegIFNα2b plus ribavirin had a higher proportion of depression than patients treated with PegIFNα2a plus ribavirin (30% vs.22%, P = 0.003) [12].

This study has several limitations. The first is the lack of a control group. As such, drug-induced DSD cannot be differentiated DSD due to disease progression. Second, the capability of the BDI questionnaire to access depression and DSD may not be validated. The structured MINI was used to minimize the error. Third, sexual hormone levels were not monitored, including the menstrual status of female patients. Last, the time point for evaluation did not extend to 24 weeks after peg-interferon plus ribavirin therapy to study the possible recovery of decreased sexual desire.

## Conclusions

Depressive symptoms frequently develop during PegIFN plus RBV treatment. Decreased sexual desire is also a common adverse effect, especially in male patients. In clinical practice, people planning to receive PegIFN and RBV should be counseled regarding the possibility of depression and DSD during treatment. It is imperative for physicians to recognize and manage treatment-related neuropsychiatric side effects and DSD. Prospective studies with larger case numbers are warranted to corroborate the findings.

## Abbreviations

DSD: Decreased sexual desire
non DSD: no decreased sexual desire
BDI: Beck Depression Inventory
BDI21: 21st item of the Beck Depression Inventory
